# The effectiveness of vitamin D supplementation in functional outcome and quality of life (QoL) of lumbar spinal stenosis (LSS) requiring surgery

**DOI:** 10.1186/s13018-020-01629-2

**Published:** 2020-03-24

**Authors:** Sangbong Ko, Seungbum Chae, Wonkee Choi, Jaibum Kwon, Je-Yong Choi

**Affiliations:** 1 Department of Orthopaedic Surgery, College of Medicine, Daegu Catholic University, Duryugongwon-ro 17-gil, Nam-gu, Daegu city, Korea; 2 Department of Biochemistry and Cell Biology, Kyungpook National University, Daegu, Korea

**Keywords:** Lumbar spine, Spinal Stenosis, Surgery, Vitamin D, Functional Outcome, Quality of Life

## Abstract

**Study design:**

This is a retrospective cohort comparative study.

**Background:**

Vitamin D supplementation is considered to be associated with good functional outcome. Thus, a few studies have proposed vitamin D supplementation is benefit to the functional outcome in LSS requiring surgery. The purpose of this study is to identify the prevalence of vitamin D deficiency in patients with LSS requiring surgery, and to compare the differences between the cases whether vitamin D is supplemented and vitamin D is not supplemented in terms of a QoL during postoperative 2 year.

**Methods:**

All patients with LSS who underwent surgery from March 1, 2015 to August 31, 2016 were enrolled. Among them, 61 patients with vitamin D deficiency were divided into two groups (supplemented group (A) and non-supplemented group (B)). Functional outcomes using Oswestry Disability Index (ODI) and Rolland Morris Disability Index (RMDQ) and QoL using SF-36 were evaluated at 12-month and 24-month follow-up periods. Differences in functional score and SF-36 between the vitamin D supplemented and non-supplemented group were compared.

**Results:**

Among the total 102 patients, 78 patients (76.5%) had vitamin D deficiency. Of the 78 patients, 61 patients were included, 27 patients were group A and 27 patients were group B. There was no difference in age and 25-OHD level between the two groups (all 0 > 0.05). Group A were better functional outcomes at 2 years after surgery (*p* < 0.05). On the QoL, group A were higher score than group B from 12 month later after surgery (*p* < 0.05).

**Conclusions:**

Vitamin D deficiency was highly prevalent in LSS patients (76.5%). Assessment of serum 25-hydroxyvitamin D (25(OH)D) is recommended in LSS needing surgical intervention and active treatment vitamin D supplementation and maintenance of normal range should be considered for better postoperative functional outcome and QoL.

## Background

Vitamin D plays a role of maintaining the extracellular calcium ion level, which plays an important role in various signaling pathways related to musculoskeletal function [1]. In addition, supplementation of vitamin D has the effect to prevent bone fracture by affecting bone mineralization [2], and has been proven to prevent falls by improving muscle functions [3]. Whereas it has been reported that vitamin D is effective in the prevention of chronic diseases such as cancer, osteoarthritis, diabetes, and cardiovascular diseases recently [4], the authors do believe that the role of vitamin D deficiency may have been somewhat neglected by the scientific literature in the past decades, shifting to the extreme opposite of considering vitamin D supplementation the “panacea” which may solve every illness also seems quite irrational. Since the 1980s, it is said that sunlight induces skin cancer and promotes aging, so many people do indoor activities and use a sunscreen during outdoor activities, gradually increasing the vitamin D deficiency and causing vitamin D-related diseases such as rickets and osteomalacia [5]. Studies have recently revealed that more than half of the world’s population is experiencing vitamin D deficiency [6] and that vitamin D’s importance is re-emphasized by studies that have examined the effects of vitamin D on calcium homeostasis, prevention, and treatment of bone related diseases such as osteoporosis. It is a current trend in the USA and various countries to increase the recommended amount of vitamin D intake.

LSS is a disease with increasing prevalence mainly due to aging. It usually accompanies lower leg radiating pain (LLRP) and low back pain (LBP) and neurogenic claudication. Although there has been a study of the prevalence of vitamin D deficiency in patients with LSS [7, 8], there has not been any study on the prevalence of vitamin D deficiency in patients with severe LSS requiring surgery and any study on the evaluation of postoperative functional outcome measures designed to estimate pain and QoL after supplementation of vitamin D for patients of vitamin D deficiency.

The purpose of this study is to study the prevalence of vitamin D deficiency in patients with LSS who require surgery, and to compare the differences between the case where vitamin D is supplemented for patients with LSS who require surgery and the case where vitamin D is not supplemented for patients with LSS who require surgery in terms of a functional outcome and a QoL.

## Methods

### Patient population

After obtaining approval from our Institutional Review Board (IRB) (approval number: CR-19-110), informed consent was waived from all participants. The vitamin D level of all the 102 patients who underwent surgery for LSS from March 1, 2015 to August 31, 2016 was investigated. The sample size showing power of 0.8, effect size of 0.91, and α error of 0.05 with ODI score difference of 15% was 20 for each group. Thus 40 subjects were needed for this study. The target patients were those who had chief complaints of lower leg radiating pain as well as neurogenic claudication associated with LBP and referred buttock pain. For these patients, surgery (decompression without fusion) by a spinal specialist was decided, as they showed the findings of spinal canal stenosis (midsagittal diameter of less than 12 mm), no foraminal stenosis, no instability via magnetic resonance imaging (MRI), the LSS not being successfully treated by conservative measures.

Of the 78 patients with vitamin D deficiency, 61 patients eligible for inclusion and exclusion criteria (Table 1) were divided into two groups—one group (called group A) who take vitamin D themselves and the other group (called group B) who do not take vitamin D themselves at a ratio of 1:1. Patients in group A take an intramuscular injection of 100,000 IU of vitamin D3 (D3BASE, ABIOGEN Pharma SpA, Italy) (group A) themselves but, patients in group B do not take vitamin D supplementation. Patients in both groups were informed of the side effects of the Vitamin D injection (headache, weakness, asthenia, muscle aches, anorexia, weight loss, nausea, vomiting, and constipation, etc.) and signed an agreement. All patients showed improved LBP, LLRP, and claudication compared to preoperative symptoms with statistically after surgery. So, visual analogue scale (VAS) for LBP, LLRP, and claudication was not performed.

**Table 1:** Inclusion and exclusion criteria

Inclusion criteria	
1	Patients with at least one lumbar spinal stenosis (midsagittal diameter of less than 12 mm) on MRI
2	Patients who do not have other abnormal findings of the spine (infection, fracture, tumor, etc.)
3	Patients who fully understood this study and voluntarily agreed to the written consent
4	Vitamin D deficiency group
5	Patients who underwent decompression without fusion
Exclusion criteria	
1	Patients taking more than 800 IU of vitamin D_3_ daily
2	Patients with serum calcium level greater than or equal to 10.5 mg/dl
3	Patients with hypercalciuria (spot urine calcium/creatinine ratio > 0.4)
4	Patients with malabsorption disease, lymphoma, sarcoidosis, tuberculosis, hyperparathyroidism, and celiac disease
5	Patients with Kidney stone or renal function (GFR < 30 ml/min/1.73 m^2^)
6	Patients with a problem with hepatic function
7	Patients with fasting blood sugar (> 126 mg/dl)
8	Patients who received spinal surgery in the past
9	A patient with a medication contraindicated
10	Patients with lower extremity fractures or vertebral fractures within the past year
11	Patients with psychiatric problems such as depression
12	Patients with gait problems due to other medical problems

*MRI :* magnetic resonance imaging, *GFR* glomerular filtration rate

### Functional outcome and QoL evaluation

The functional evaluation for the two randomly divided groups was the validated Korean version of the ODI (version 2.0) and the RMDQ having a score range of zero to 100. The functional evaluation was performed in the aid of the trained clinical research coordinator at 12 months and 24 months postoperatively. In addition, SF-36 questionnaire, a tool for assessing QoL, was filled out by the patient himself with the help of the same coordinator at 12 months and 24 months postoperatively.

### Measurement and supplementation of 25(OH)D

Blood samples were collected between 8:00 am and 8:30 am with patients under fasting condition, in order to reduce errors in circadian variation. Serum 25(OH)D was measured by chemiluminescence immunoassay (LIASON-XL, DiaSorin, Inc., Stillwater, Minnesota, USA). Serum 25(OH)D levels below 20 ng/ml (50 nmol/L) were indicated as deficient; 21–29 ng/ml as insufficient; and above 30 ng/ml (75 nmol/L) as normal [9]. In both groups, 5 mg of limaprost-alpha-cyclodextrin clathrate (Opalmon Tab, Dong-A ST, Seoul, Korea) was administered orally three times a day, and the medication was adjusted accordingly the degree of claudication.

### Statistical analysis

Differences in functional score and SF-36 between the vitamin D supplemented and non-supplemented group were compared using a Mann Whitney *U* test. The statistical analysis was performed using the SPSS software version 19.0 for windows (SPSS, IBM Corporation, Armonk, NY, USA). We considered *p* ≤ 0.05 as statistically significant. In addition, between-group differences in terms of VAS scores and functional outcomes were analyzed using Mann-Whitney *U* test, the power of result in the VAS score difference and functional outcome was determined with G power 3.1.

## Results

### Epidemiological results

The average age of 102 patients (34 males, 68 females) was 68.26 ± 9.25 years. The average age of patients with vitamin D deficiency (a total of 78, 27 male and 51 female) was 68.94 ± 9.04 years, and the average age of patients without vitamin D deficiency (a total of 61, 23 male and 31 female) was 68.88 ± 8.42 years. There was no statistical significance between the two groups (*p* = 0.715). Out of the group of vitamin D deficiency, the average age of the group A (total 27, 11 male and 16 female) was 69.44 ± 2.56 years, and the average age of the group B (total 27, 12 male and 15 female) was 68.47 ± 2.42 years. There was no statistical significance between the two groups (*p* = 0.764). The average 25(OH)D result of 78 patients with 25(OH)D deficiency was 12.87 ± 4.41 (range, 4.07 to 19.5). The preoperative average 25(OH)D values were 10.44 ± 1.02 in group A and 10.97 ± 1.00 in group B, respectively, and there was no statistical significance (*p* = 0.657) (Table 2). The postoperative average 25(OH)D values were 31.23 ± 1.09 in group A and 16.24 ± 3.21 in group B at postoperative 12 months, and 32.14 ± 2.12 in group A and 17.29 ± 4.38 in group B at postoperative 24 months, respectively, and there were statistical significance (*p* < 0.05) (Table 2).

**Table 2:** Epidemiological results

	Vitamin D non-deficient		Vitamin D deficient		*p* value
Number of patients (male/female)	24(7/17)		78(27/51)		
			Group A	Group B	
			27(11/16)	27(12/15)	
Average age	68.88 ± 8.42		68.94 ± 9.04		0.715
			Group A	Group B	
				68.47 ± 2.42	0.764
25(OH)D (preoperative)			10.44 ± 1.02	10.97 ± 1.00	0.657
25(OH)D (postop. 12 month)			31.23 ± 1.09	16.24 ± 3.21	0.002^*^
25(OH)D (ostop. 24 month)			32.14 ± 2.12	17.29 ± 4.38	0.003^*^
Level of surgery		L3-4	1	1	
		L4-5	21	20	
		L5-6	5	6	

*25(OH)D* 25-hydroxyvitamin D, *Postop*. postoperative

*Statistically significant with *p* < 0.05

### Results of functional outcome and QoL

The preoperative ODI was 59.88 ± 4.06 in group A and 57.65 ± 3.31 in group B, showing no statistically significant difference (*p* = 0.683). The preoperative RMDQ was 14.75 ± 1.07 in group A and 15.18 ± 0.84 in group B, showing no statistical significant difference (*p* = 0.683). The ODI after 12 months from the surgery was 22.00 ± 2.70 in group A and 28.12 ± 2.10 in group B, showing statistical significant difference (*p* = 0.034). The RMDQ was 6.06 ± 0.86 in group A and 7.71 ± 0.95 in group B, having no difference (*p* = 0.217). The ODI after 24 months from the surgery was 11.00 ± 1.52 in group A and 20.94 ± 1.86 in group B, indicating a better result in group A (*p* = 0.0002). The RMDQ was 3.06 ± 0.38 in group A and 4.94 ± 0.82 in group B, indicating a better result in group A (*p* = 0.110) (Table 3).

**Table 3:** Result of functional outcome

	ODI		*p* value	RMDQ		*p* value
	Group A	Group B		Group A	Group B	
Preoperative	59.88 ± 4.06	57.65 ± 3.31	0.683	14.75 ± 1.07	15.18 ± 0.84	0.683
12 month	22.00 ± 2.70	28.12 ± 2.10	0.034^*^	6.06 ± 0.86	7.71 ± 0.95	0.217
24 month	11.00 ± 1.52	20.94 ± 1.86	0.0002^*^	3.06 ± 0.38	4.94 ± 0.82	0.110

*ODI* Oswestry Disability Index, *RMDQ* Rolland-Morris Disability Questionnaire

*Statistically significant with *p* < 0.05

As for the QoL using SF-36 questionnaire, preoperative mental component score (MCS) was 38.19 ± 3.49 in group A and 29.71 ± 4.53 in group B, showing no statistical significant difference (*p* = 0.136). Preoperative physical component score (PCS) was 30.90 ± 3.20 in group A and 26.12 ± 3.66 in group B, showing no statistical significant difference (*p* = 0.168). MCS after 12 months from the surgery was 63.71 ± 5.38 in group A and 43.17 ± 4.79 in group B. The PCS of the same period was 60.27 ± 5.11 in group A and 36.95 ± 4.42 in group B, indicating that A group shows better MCS and PCS results (*p* = 0.005, p = 0.001). The MCS after 24 months from the surgery was 82.61 ± 2.19 in group A, 52.06 ± 5.43 in group B, and the PCS of the same period was 70.05 ± 3.41 in group A and 51.65 ± 4.64 in group B, respectively. The results of MCS and PCS were better in group A (*p* < 0.005) (Table 4).

**Table 4:** Result of QoL

	SF-36 MCS		*p* value	SF-36 PCS		*p* value
	Group A	Group B		Group A	Group B	
Preoperative	38.19 ± 3.49	29.71 ± 4.53	0.136	30.90 ± 3.20	26.12 ± 3.66	0.168
12 month	63.71 ± 5.38	43.17 ± 4.79	0.005^*^	60.27 ± 5.11	36.95 ± 4.42	0.001^*^
24 month	82.61 ± 2.19	52.06 ± 5.43	< 0.005^*^	70.05 ± 3.41	51.65 ± 4.64	< 0.005^*^

*MCS* mental component score, *PCS* physical component score

*Statistically significant with *p* < 0.05

## Discussion

Jacques et al. [10] asserted that homebound elderly persons who have no specific disease and stay at home have high prevalence of vitamin D deficiency and thus, vitamin D supplementation is required. Kim et al. [8] reported a high prevalence of vitamin D deficiency in elderly patients who find substantial difficulties to go outside due to claudication attributable from LSS. Therefore, they said that even supplementation of vitamin D alone can maintain a stable health status by increasing muscle strength to some extent and keeping balance of muscle strength [3, 11], and Bischoff-Ferrari [12] stated that vitamin D supplementation would reduce the falling accidents of the elderly by about 22%.

Although healthy adults are not deficient through sufficient meals and sunlight, others are recommended that 25(OH)D level in serum maintains 20 ng/ml or more. However, Tangpricha et al. [13] reported that vitamin D deficiency occurs even in healthy young adults; 11% in summer and 26% in winter. Kim et al. [8] reported that the elderly patients with LSS showed increasing prevalence of vitamin D deficiency, and about 74.3% of them had vitamin D deficiency. The authors of this paper also stated that a prevalence of 76.5% (78/102 patients) was exhibited in patients with LSS who require surgery. The authors supposed the cause of vitamin D deficiency was the walking problems due to intermittent neurogenic claudication, and then restriction of going out, but did not make an accurate investigation.

For patients with LSS, surgical treatment has better results than palliative treatment in terms of physical function, pain, and QoL [14]. As described by Kim et al. [7], decompression surgery for patients with LSS showed elevated vitamin D levels, and after 1 year, the levels increased from 11.1 to 14.2 ng/ml in the depletion group and 23.2 to 23.1 ng/ml in deficiency group, but neither of the two groups recovered normal levels. The authors of this paper believed that vitamin D supplementation for the vitamin D deficient group before surgery would be reasonable, and concluded that supplementation of vitamin D for the vitamin D deficient group before surgery would result in better functional result of spine and improve QoL.

In our results, ODI after 12 month surgery was improved in vitamin D supplementation group. RMDQ was not different between preoperative and all postoperative periods. However, both SF-36 MCS and SF-36 PCS improved in vitamin D supplementation group from 12 month after surgery. Although there is a growing body of literature suggesting a possible negative influence of preoperative vitamin D deficiency upon surgical outcomes in spine surgery, the vast majority of the literature focuses on the causative link between vitamin D deficiency and pseudoarthrosis [15] or new vertebral fractures [16].

The effect of vitamin D on the functional outcome and QoL of patients with LSS has not been elucidated but may be deduced from several studies. There is much debate about the pain relief effect of Vitamin D. Although it is a study of idiopathic chronic LBP, Cannell et al. [17] have reported on the pain relief effect of vitamin D. Helde-Frankling et al. [18] have commented on the pain reducing effect of Vitamin D, Cakar et al. [19] said that in a cohort of 149 patients, the authors found that serum vitamin D concentration was not associated with knee pain in patients with osteoarthritis, and Heuch et al. [20] said that after analyzing a data set including 1685 individuals with LBP and 3137 controls without LBP, the authors found no association between vitamin D status and risk of LBP. In addition, it appears that vitamin D may have an effect to enhance mood, but there are not sufficient studies regarding accurate mechanism [21]. The results of this study showed that both ODI and the QoL after 12 months were improved. In this regard, supplementation of vitamin D would improve functional outcome and enhance QoL in a long-term perspective, if the normal level of vitamin D is maintained. However, authors of this study could not figure out the mechanism.

The limitations of this study are as follows: First, there was a lack of research on socio-demographic characteristics. As Kim et al. [8] pointed out, there was a lack of consideration of level of education [22], season [23] as well as medical comorbidity, urban residence, and sunlight exposure. However, this study evaluated serial changes in the same patient. There was no significant difference in the degree of education level, seasonal exposure, medical comorbidity, urban residence, and sunlight exposure. Second, there are many studies on LSS associated with various chronic diseases such as diabetes, hypertension, chronic obstructive pulmonary disease, and gout, but there has been no study on mutual causal relationship [24]. In addition, several authors have shown that vitamin D itself has the effect of preventing the deterioration of the physical function of a patient by preventing the chronic disease such as cancer, osteoarthritis, diabetes, and cardiovascular diseases [4, 7, 25, 26]. This study showed a drawback that errors due to factors related to chronic diseases of patients who needed surgery could not be completely eliminated. Third, only the results of improvement of functionality and QoL were obtained. Fourth, the number of patients in each group was too small (27 patients in group A and 27 patients in group B) to analyze the mechanism and related factors, although this was a randomized method. In addition, there was limited data about how long was vitamin D administrated before surgery and how long did it take to normalize the vitamin D serum levels postoperatively.

## Conclusion

The prevalence of vitamin D deficiency in patients with LSS requiring decompression surgery was 76.5%. Although the increase in vitamin D levels in a certain level may be expected if the gait improves after decompression surgery, it seems necessary to maintain 25(OH)D level in serum by checking vitamin D deficiency before surgery to enhance functional outcome of spine or improve QoL of a corresponding patient in a long-term perspective.

## Data Availability

Yes

## Abbreviations

QoL: Quality of life
LSS: Lumbar spinal stenosis
ODI: Oswestry Disability Index
RMDQ: Rolland Morris Disability Index (RMDQ)
25(OH)D: 25-hydroxyvitamin D
LLRP: Lower leg radiating pain
LBP: Low back pain
MRI: Magnetic resonance imaging
VAS: Visual analogue scale
MCS: Mental component score
PCS: Physical component score
